# Bayesian Inference on the Effect of Density Dependence and Weather on a Guanaco Population from Chile

**DOI:** 10.1371/journal.pone.0115307

**Published:** 2014-12-16

**Authors:** María Zubillaga, Oscar Skewes, Nicolás Soto, Jorge E. Rabinovich, Fernando Colchero

**Affiliations:** 1 Centro de Estudios Parasitológicos y de Vectores (CEPAVE, CONICET- CCT- La Plata, UNLP), Universidad Nacional de La Plata, La Plata, Argentina; 2 Department of Mathematics and Computer Science (IMADA) and Max-Planck Odense Center on the Biodemography of Aging (MaxO), University of Southern Denmark, Odense, M 5230, Denmark; 3 Departamento de Ciencias Pecuarias, Universidad de Concepción, Chillán, Chile; 4 Departamento de Protección de Recursos Naturales Servicio Agrícola Ganadero (SAG), XII Región, Chile; Université de Sherbrooke, Canada

## Abstract

Understanding the mechanisms that drive population dynamics is fundamental for management of wild populations. The guanaco (*Lama guanicoe*) is one of two wild camelid species in South America. We evaluated the effects of density dependence and weather variables on population regulation based on a time series of 36 years of population sampling of guanacos in Tierra del Fuego, Chile. The population density varied between 2.7 and 30.7 guanaco/km^2^, with an apparent monotonic growth during the first 25 years; however, in the last 10 years the population has shown large fluctuations, suggesting that it might have reached its carrying capacity. We used a Bayesian state-space framework and model selection to determine the effect of density and environmental variables on guanaco population dynamics. Our results show that the population is under density dependent regulation and that it is currently fluctuating around an average carrying capacity of 45,000 guanacos. We also found a significant positive effect of previous winter temperature while sheep density has a strong negative effect on the guanaco population growth. We conclude that there are significant density dependent processes and that climate as well as competition with domestic species have important effects determining the population size of guanacos, with important implications for management and conservation.

## Introduction

To understand wildlife population dynamics it is important to identify how they are affected by environmental factors and density dependence (DD) processes; it is also critical for conservation and wildlife management, particularly in those species living in extreme environments. Although several studies have shed light on these issues for several species, e.g., wildebeest [1], white-tailed deer [2], and elands and impalas [3], still little is known about the mechanisms that regulate the populations of groups such as the wild South American camelids. This is the case of the heavily exploited guanaco (*Lama guanicoe*) populations, that have diminished significantly during the last century because of grazing conflicts with a sheep-based society, and overhunting; in only three years between 1977 and 1980 around 140,000 guanacos were officially exported from Argentina mainly to eight countries in North America and Europe [4]. Due to decades of hunting, the guanaco's distribution has been reduced by 75% in Chile and Peru, and by 60% in Argentina [5]. Although the guanaco is categorized as least concern by the IUCN Red List [6], to tackle the dramatic decline of guanaco populations this species has been included in Appendix II of CITES [7]. A sustainable management of guanacos requires understanding the processes that regulate their populations [8].

In nature all species are subject to checks and balances that limit their population growth; these result from the action of different processes that restrict population size and/or geographic distribution; such processes can be density-stabilizing or density-limiting [9]. The former is of a biotic nature and depends on the interaction between individuals of the same or different species; the latter is independent of population size. Stabilization results from density-dependence: its regulatory effect varies in intensity with the size or density of the population itself. However, not all DD factors are density-stabilizing. Therefore, analyses that ignore the effects of density can produce misleading guidelines for population management if DD actually occurs. In addition, when the maximum sustained harvest criterion is used for wildlife management, it is necessary to estimate the population size at which population growth rate is maximum, but this estimation depends on the shape of the DD function [10],[11].

In addition, population growth can be affected by the interaction between density dependence environmental variables; the strength of DD and of environmental variables on the regulation of the desert bighorn sheep population (*Ovis canadensis*) were explored using Bayesian state space models [12]; those results showed that the population's growth rate was dominated by density-dependence and, although drought had a secondary effect, it was still relevant to the population dynamics. In terms of the persistence of the population, the results of [12] emphasize that a more variable environment is less threatening than one in which the mean conditions become harsher.

Although there are various studies of density-dependence processes in mammals in general [13],[14], and in ungulates in particular [15], [16],[17],[18] there are very few study in wild South American camelids that analyze the effect of density dependency on population growth. To our knowledge, the only studies in this taxonomic group have been carried out in populations of vicuña (*Vicugna vicugna*) [19], [20], [21], and guanacos, the latter focusing on the effect of density-dependency over some demographics parameters [22], [23], [24]. However, in a previous study [25] we evaluated the effect of density, the impact of sheep density and of weather variables on the growth rate of a guanaco population growth in Chile, using a multiple linear regression analysis; the results showed that only population size was a statistically significant predictor variable. However, many studies on ungulates [26] [27] have shown that population growth models that explicitly account for the Markov chain structure of population dynamics processes provide accurate predictions of the effect of climate and density-dependence on population growth rates. Therefore, we considered of interest to use population growth models on the same guanaco population that we previously analyzed [25], while accounting for the effect of weather variables and sheep densities.

Although the guanaco has a high economic value as a commercial species (for its hair, meat and skin), it is considered a pest by sheep ranchers because it has been popularly considered as a potential competitor for forage and water. Present management programs are incipient and markets are still not fully developed, so rigorous evaluations of density-dependence and environmental impacts in these populations are important not only for scientific purposes, but also for the design of sustainable management plans to avoid uncontrolled hunting by ranchers (unpublished report to the Secretary of Wildlife of the Province of Chubut, Argentina). Here we used a Bayesian model to determine the strength of density dependence regulation on the Tierra del Fuego guanaco population as well as we tested the influence of winter temperature and average annual precipitation on the population's growth rate.

## Materials and Methods

### Ethics statements

The present study did not require the capture or handling of animals. Data for this study is based on public information provided by the Chilean State Wildlife Control Service or obtained as direct observations by the authors. Field observations were made from public roads and did not require special permits, and when in private lands appropriate permission was granted to the authors of this study by private land owners.

### Study area

**Figure 1:**
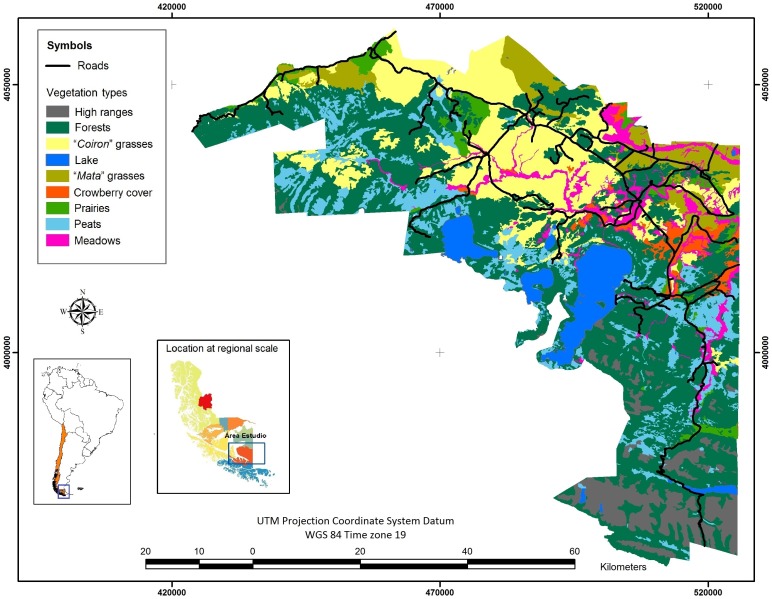
Study area. Location of the study area and distribution of vegetation types (modified from [28]).

This study was carried out on a guanaco population of the “Cameron” ranch (53.9° S, 69.3° W) located in the Southern region of the Tierra del Fuego Island, Chile. The ranch covers an area of the 2000 km^2^, and its altitude range is 0 – 300 m.a.s.l. The region is a mosaic of the steppe (“*pampa*”) and forest biomes; the latter is composed by deciduous forests dominated by lenga (*Nothofagus pumilio*) and ñire (*N. antartica*). The steppe is composed of several species of the genera *Stipa, Festuca* and *Rytidosperma* (“*coirón*” grasses), of the genera *Chiliotrichium, Berberis, Baccharis*, and *Lepydophillum* (“*mata*” grasses), of the genera *Empetrum*, *Baccharis*, *Discaria*, *Pernettya, Bolax* and *Azorella* (“*murtilla*” grasses), of the genera *Holcus, Dactylis, Trifolium*, and *Agrostis* (meadow grasses); of the genera *Poa, Azorella, Hordeum, Samolus, Festuca*, as well as species of Juncacea and Cyperacea (“*vega*” grasses, the most fertile type of grassland, found in poorly drained and more humid, areas), and of the genera *Carex, Empetrum, Marssippospermum, and Sphagnum*) (peat bogs)[28]. Fig. 1 shows the distribution of the different types of vegetation in the study area, although some of the species have recently been seriously reduced by sheep, the dominant domestic species, with densities that have fluctuated in the last decades between 11 and 23 sheep/km^2^.

In Tierra del Fuego island guanacos are not preyed upon by pumas (*Puma concolor*) as it happens in the continent [29], and where also some culpeo foxes (*Lycalopex culpaeus*) occasionally prey upon newborn guanacos [30]. In non-forested areas (the Patagonian steppe), from the east of the Andes to the Atlantic coast, the climate is characterized by an average annual precipitation of 200–400 mm (in the Cameron ranch itself the average is 370 mm/year), while in the forested areas the average precipitation fluctuates around 400–600 mm per year [31]. The average annual NDVI (Normalized Differential Vegetation Index), which is a dimensionless value in the range 0–1, and used as an indicator of primary productivity, is around 0.56, but highly seasonal (between 0.6–0.7 in the summer period of December-April, and about 0.4–0.5 in the winter period of June-August) (unpublished report to the Secretary of Wildlife of the Province of Chubut, Argentina). Given a precipitation of 370 mm/year the estimated above-ground net primary productivity (ANPP) was 1600 dry matter kg/ha/year [32].

In South America there are only two species of wild camelids: the vicuña and the guanaco; the latter ranges from Northern Peru through Chile, and across Patagonia to southern Argentina and Chile, reaching Tierra del Fuego; it occupies a wide variety of habitats from hardpan deserts to scrublands to grasslands, from sea level to nearly 4500 m on the Andes' mountain range [33]. The guanaco mating system is a resource defense polygyny, where five major social units have been recognized: (i) family groups (one adult dominant male, various adult females and several young), (ii) solitary territorial males (defending a territory but usually without females), (iii) male groups (non-territorial males), (iv) female groups (adult females with or without young), and (v) mixed groups (individuals of both sexes and all ages) [24]. From spring to autumn adult females are socially and spatially segregated from non-territorial males, and parturition occurs in the summer, with females producing one offspring after about 11.5 months of gestation [24].

### Guanaco population sampling

Guanacos were counted for 34 consecutive years between 1977 and 2012 (the exceptions were years 1986 and 1996), using the transect method with a variable width from 1977 to 2000, and with a fixed width band from 2001 to 2012 (the latter with a maximum of 500 m to each side of the transect) [34], [35], [36], [37], [38]. The sampling period was carried out in autumn of each year and lasted approximately 7 days, between 10:30 and 19:00 h, with two observers in each of two 4×4 vehicles going over the main, secondary and local roads at a maximum speed of 40 km/h. Each road was covered only once and particular care was taken to avoid potential duplication of counts at the road intersections. In addition to individual guanaco counts, the following were recorded: weather conditions, time, distance (km) from the starting point, GPS coordinates, observation distance from the transect (m), an estimate of the angle to the animal's position, and –when the animals were observed in groups– the number of individuals, the type of social group, and its structure (sex, and the age class: newborn, juvenile, and adult). The road network and all geo-referenced observations were processed with the Arc View 9.3 Geographical Information System (GIS), and transferred using program Map Source. The cartography was kindly provided by the Chilean “*Servicio Agrícola y Ganadero*” (SAG). The area effectively surveyed in each sampling period was around 420 km^2^ (the average length of the transects was 420 km and the width band was 1 km) which represent about 20% of the area under study; despite the roads were not randomly distributed, as the guanaco population is well structured at the period of the year when sampling was carried out (particularly the family groups), it was assumed that the guanaco density in the area surveyed is representative of the whole ranch. The population size was estimated as given in [28] which was based in the method described and implemented in [31]; more details on the statistical analyses of the sampling can be found in [25].

### Sheep population

Sheep population was based on a time series obtained from Soto (personal communication); only eight years of data were available (1980, 1985, 1990, 1995, 2000, 2005, 2008 and 2011); as the sheep population change between years was relatively smooth, we interpolated linearly between two consecutive data points to generate a complete time series of 36 years.

### Environmental covariates

We evaluated the effects of density dependence and of environmental covariates on guanaco population dynamics; the environmental covariates considered were: annual precipitation and average winter temperature (months of June, July and August), and 25 years of data (1977–2002) were from the CRU TS 2.1 database, compiled by the Tyndall Centre, Climatic Research Unit, School of Environmental Sciences of the University of East Anglia, United Kingdom (http://www.cru.uea.ac.uk/cru/data/hrg.htm). Because the CRU data ended in 2002, we completed that time series for 2003 to 2012 with data from Punta Arenas (Chile), the closest meteorological station to the Cameron ranch; this data was downloaded from the Internet site of the Meteorological Service of Chile (http://www.meteochile.gob.cl/).

For each of these covariates, at a given time *t* we calculated the anomaly for a given value *v_t_* as
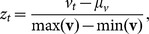
where *µ_v_* is the empirical mean of the weather series represented by the vector **v,** and *z_t_* is the resulting anomaly.

In order to make the parameter estimates comparable, we also re-scaled the sheep counts as *z_t_*  =  *v_t_*/max(*v_t_*), where *v_t_* is the estimate of the number of sheep in year *t*.

### Population analysis

We evaluated the effect of density-dependence and environmental covariates in the regulation of the Tierra del Fuego guanaco population by the method of the “direct density-dependence” [39] using a Bayesian state-space framework [40].

Here we assumed that population counts were obtained with errors, and thus we define the random variable *O_t_* for counts on a population at a given time *t*. Furthermore, because the actual population size is unknown, we define the random variable *N_t_* for the population size at a given time. The conditional probability of an observation of counts at time 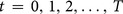, where *T* is the last year of the study, is given by the negative binomial observation model
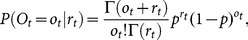
(1)where *p* is the probability that a single individual is detected and *r_t_* is a size parameter. The theoretical mean of the count random variable is E[*O_t_* | *n_t_*]  =  *n_t_*, and 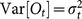. Following Linden and Mantyniemi's [41] notation, we have that 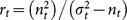 and the probability is given by 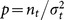, which simplifies the size parameter to *r_t_*  =  *n_t_*/(1/p - 1) and the variance is 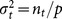. We used the negative binomial model to account of potential overdispersion in the counts [42] [41].

On the other hand, the conditional probability for a population size *N_t_* is given by the Poisson process model
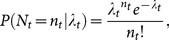
(2)where the expected value of the population size at time *t* is given by *E*[*N_t_* | *λ_t_*]  =  *λ_t_*. The process model, this is the expected population size at *t* >0, is a function of the expected population size at *t* - 1, and is given by the population growth function
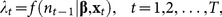
(3)where **β** is a vector of parameters to be estimated and **x** *_t_* is a vector of covariates. The joint likelihood of the vectors **o** and **n** of observations and population sizes, respectively, is given by
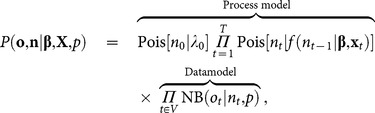
(4)where *V* is the subset of years for which counts were obtained and **X** is the *T*×*k* design matrix containing the covariates for all years where *k* is the total number of parameters in vector **β**. The full posterior for all the unknowns is given by
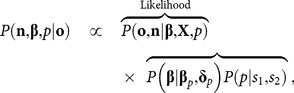
(5)where **β** *_p_* is a vector of mean prior parameters and and **δ** *_p_* is a prior covariance matrix for the growth parameters in equation 3, while *s*_1_ and *s*_2_ are priors for the probability *p*.

We used a Markov chain Monte Carlo (MCMC) algorithm that combined Metropolis sampling [43] [44] for the population growth parameters **β** and the latent population sizes **n**, and direct sampling for the probability *p* in the data model [44]. We used normal priors for the population growth parameters such that **β** ∼ N_k_(**β** *_p_*, **δ** *_p_*), and used a conjugate beta prior for *p* such that
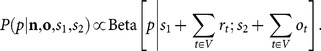
(6)


We ran ten parallel chains for 210,000 iterations, with a burn in of 10001 and thinned the resulting sequences every 200 steps to reduce serial autocorrelation. Each chain was started from different initial values. We used potential scale reduction as proposed by Gelman *et al* [45] and calculated potential scale reduction to evaluate convergence.

Within the MCMC sampler we also constructed predictive distributions for the observations by sampling a new set of predictive **o**, noted 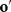, integrated across the posterior densities of the parameters
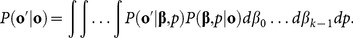
(7)


This integral is evaluated numerically from which the expected predictions are calculated as
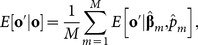
(8)where 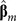 and 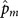 are the estimated parameters at step *m*. With (8) we predicted **o'** at all the remaining *m* steps of the MCMC for a total of *M* = 10,000, and then calculate the mean and quantiles for each observation to construct predictive intervals. We used these predictive distributions for model selection by calculating predictive loss (*D_m_*) [46], which combines a measure of model goodness of fit based on the error sum of squares
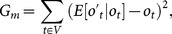
(9)with a measure of model dispersion (or penalty term), measured as the predictive variance



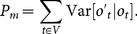
(10)The model with the lowest *D_m_  =  G_m_ + P_m_*, is then selected. This approach has advantages over more traditional methods such as the use of Akaike information criterion (AIC) in that it does not rely uniquely on a measure of goodness of fit and a penalization term, but it evaluates the predictive capabilities of the model and penalizes over-fitting based on the spread of the predictive distributions [44]. Thus, relevant covariates that could be automatically discarded under a traditional method can still be considered as long as their effect is significant.

### Models tested

To evaluate if the population of guanacos in Tierra del Fuego is regulated by density dependence, precipitation and winter temperature, we defined function *f* (.) in equation 3 as a discrete exponential growth function of the form
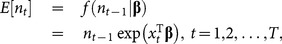
(11)where **β**  =  {*β*_0_, …, *β_k_*_-1_} and **x** *_t_* ^T^ is the transposed vector of covariates. We tested a range of nested models, starting with a simple exponential growth model of the form *E*[*n_t_*]  =  *n_t_*_-1_ exp[*β*_0_], where *β*_0_ corresponds to the intrinsic rate of population growth. This simplest model implies that the population is not regulated by density dependence. Covariates can be included as
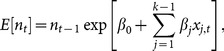
(12)where *x_j,t_* is the *j* ^th^ environmental covariate. To estimate the effect of density dependence we extended equation (12) based on the Ricker population growth model, which takes the form
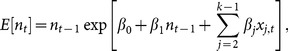
(13)where *β*_1_ is the parameter that estimates the intensity of density dependence on the population growth.

All statistical analyses were performed in the open source statistical software R [47].

## Results

S1 Table shows the time series of guanaco data for the 36 years sampled (years 1986 and 1996, with no available data, were linearly interpolated), and the values of the climatic variables.

### Bayesian analysis of density-dependence

**Figure 2:**
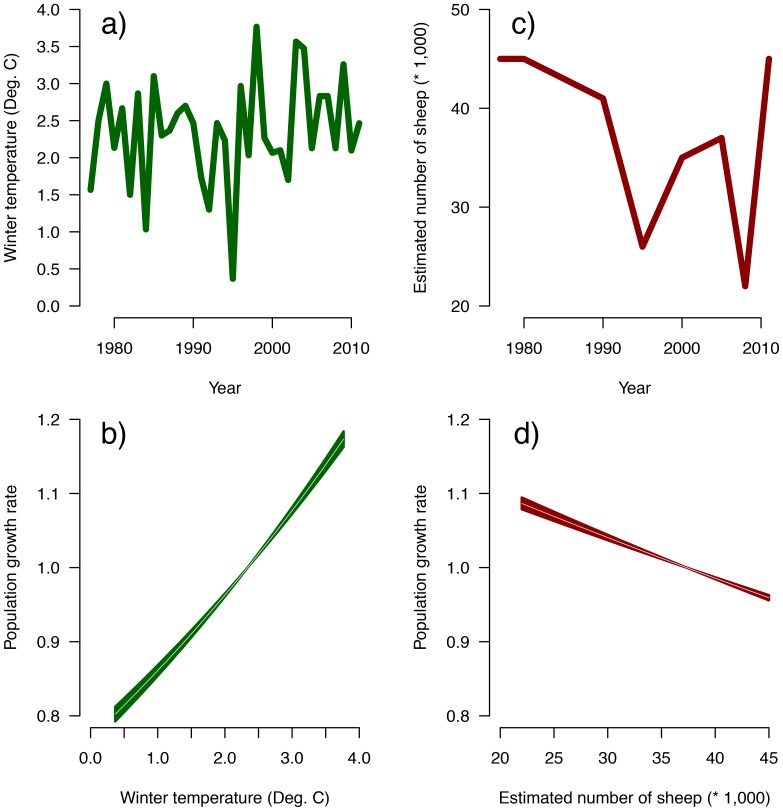
Effect of annual precipitation and sheep numbers on the guanaco's expected population growth rate. a) Average winter temperature (°C) during the study; b) effect winter temperature on the guanaco population's growth rate; c) yearly estimated number of sheep in the study area; d) effect of the number of sheep on the guanaco population growth rate. The population growth rate was calculated as *λ* =  exp(*β*_0_ + *β*_1_ K + *β*_2_ T + *β*_3_ S), where K is the carrying capacity, T are the values of winter temperature, and S are the values for the estimated number of sheep. The width of the polygons in b) and d) corresponds to the 95% credible intervals.

**Figure 3:**
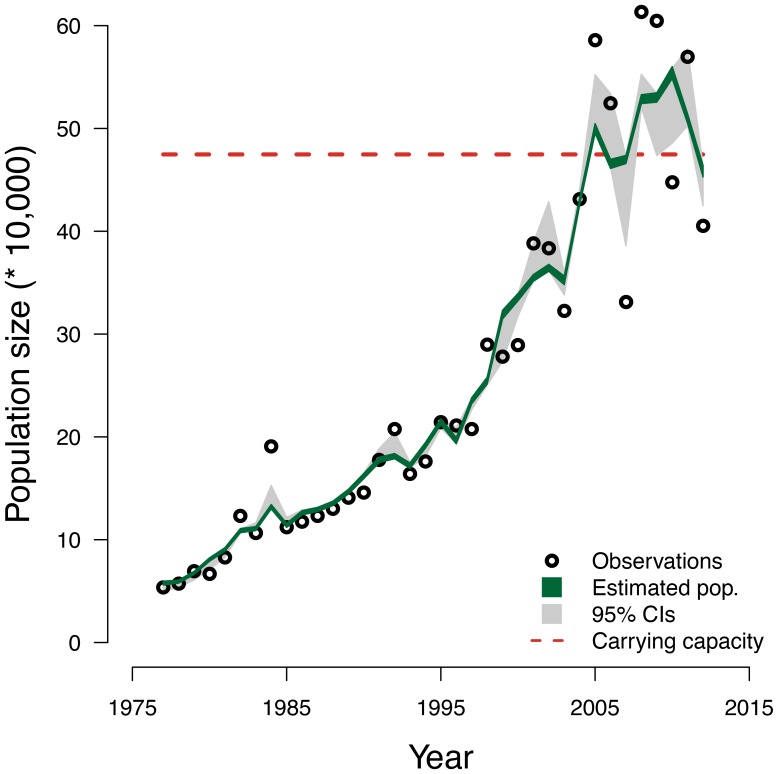
Predicted population size as a function of winter temperature, sheep density and density-dependence. The green polygon shows the predicted population sizes and the grey polygons are the 95% credible intervals for the predicted population sizes.

The model with the lowest predictive loss included density dependence, winter temperature and sheep densities (Table 1), where the conditional posterior densities of all three parameters did not include 0 (i.e. their effect was significantly different from 0; S2 Table). All the models tested that did not include density dependence (i.e. simple exponential growth models) had predictive loss values higher than the worst Ricker model. The exponential model had a deviance almost 16% higher than the Ricker model. The selected model implies that there is a significant density-dependent effect and that population growth increases positively with winter temperature but declines with increasing sheep densities (Fig. 2). The average carrying capacity during a given time, *K*, is given by
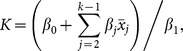
where 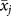
is the average value of the *j* ^th^ covariate within the interval of time of interest. For the last six years the estimated *K* is 46,563 (±15,283) individuals (about 23 guanaco/km^2^). As expected, this value is strongly affected by the density of sheep in the area, for instance, in the last year; the number of sheep was estimated at about 45,000 individuals. At this number, the carrying capacity of the guanacos drops to an estimate of *K* = 29,359 (±16,329). Fig. 3 shows the observed population size, the estimated population size with its 95% CI, and the estimated carrying capacity.

**Table 1:** Model fit (as measured by the predictive loss) of the guanaco population growth models.

Model	Goodness of fit	Penalty	Deviance
DD + Temp + Sheep	747042316.4	2588292.694	749630609.1
DD + Sheep + Temp × DD	748808123.7	2614802.017	751422925.7
DD + Temp + Precip + Sheep	751283742.7	2674436.853	753958179.5
DD + Sheep + Temp × DD + Precip × DD	753299329.9	2702261.732	756001591.6
DD + Temp × DD	782831094.6	2558115.904	785389210.5
DD + Temp	788181401.3	2587368.34	790768769.6
DD + Temp + Precip	791766848.7	2618544.645	794385393.3
DD + Temp × DD + Precip × DD	793801071.9	2639064.723	796440136.6
DD + Sheep + Precip × DD	817856927.4	2605420.646	820462348
DD + Sheep	820229740.3	2550647.293	822780387.6
DD + Precip + Sheep	823431071.4	2601587.578	826032659
DD + Precip	841143533.6	2511229.804	843654763.4
DD + Precip × DD	844055704.3	2545798.918	846601503.3
DD	854413880.7	2501355.587	856915236.3
Temp	873204686.7	2557085.83	875761772.6
Precip	878017164.2	2523181.168	880540345.4
Sheep	882878856.8	2526847.302	885405704.1
Temp × DD	883300197.6	2594072.115	885894269.7
Precip × DD	891135256.2	2553360.278	893688616.5
Temp + Precip	893374974.7	2606019.758	895980994.4
Temp + Sheep	893617459.1	2600458.55	896217917.6
Precip + Sheep	899859493.5	2489580.89	902349074.4
Temp × DD + Precip × DD	902399153.6	2550110.373	904949264
Sheep + Temp × DD	903200592.5	2560516.426	905761108.9
Sheep + Precip × DD	906910271.5	2476583.175	909386854.7
Sheep + Temp × DD + Precip × DD	909330405.9	2569412.91	911899818.8
Temp + Precip + Sheep	916053078.8	2486596.4	918539675.2

The variables tested were: density dependence (DD), average winter temperature (Temp), sheep density (Sheep) and annual precipitation (Precip). The “best” model was chosen as the model with the lowest deviance (DD + Temperature + Sheep).

## Discussion

The results of our Bayesian analysis show that the population of guanacos in Tierra del Fuego, Chile, is under strong density dependent regulation, and that it has already reached its carrying capacity (K = 46,563 individuals). In addition, we found a positive relation between population growth rate and winter temperature (Fig. 2). Moreover, we found a strong effect of the number of sheep in the area, where a marked increase in their numbers can dramatically limit the availability of resources for the guanacos.

A study similar to our present analysis was carried out with the elk [48], and the authors concluded that a density-dependence model that incorporates climatic covariates shows a better fit than without considering them. Our results are consistent with these results where winter temperature has a positive effect on the guanaco population growth.

In particular, our results suggest that the guanaco population of the Cameron ranch, Tierra del Fuego, Chile, is presently being regulated near its carrying capacity by a density-dependent process. These results are consistent with those obtained for other species of ungulates such as white-tailed deer (*Odocoileus virginianus*), elk (*Cervus elaphus*), desert bighorn sheep (*Ov. canadensis nelsoni*), mule deer (*0d. hemionus*), desert mule deer (*0d.h. crooki*) caribou (*Rangifer tarandus*) and bison (*Bison bison*) [49].

A logistic model was used to fit 31 years of vicuñas data and found a significant effect of rainfall on population growth, and that rainfall had its highest significant effect with a time lag of 4 years [21]. Thus, our results are congruent with those of the vicuñas that may allow for a preliminary generalization: South American camelid populations are regulated by density dependence while also being affected by environmental variables.

A study on a 17-years population time series on vicuña in Chile [20] estimated that the population was stationary (i.e. the per capita annual population growth rate was equal to 1). They performed a simple linear regression between population size and growth rate and concluded that there was a clear density-dependence effect; however, the large scatter of the data in the relationship between density and the population growth rate can be taken as an indication that density-independent factors also play an important role in population change. Similarly, an analysis was carried out by means a multiple linear regression in the same guanaco population of Cameron Ranch that we studied here. The analysis included the population growth rate as dependent variable and guanaco population size, sheep population size and the same weather covariates as independent variables [25]. The results suggested that the population size was the only statistically significant predictor. In comparison with the results of Zubillaga *et al* [25] the present study shows that, in addition to population density, average winter temperature and sheep densities are also significant determinants of the guanaco population growth rate. The differences in the conclusion of both analyses may be in part due to the fact that our previous approach can generate conclusions that differ substantially from the current state-space model [50]. In the current analysis we used a Markov Chain structure to model changes in population growth, while we accounted for measurement errors that were not considered in the previous analysis. Nonetheless, more research needs to be carried out to determine the exact mechanisms that relate population growth to winter temperature in the previous year.

In a study on a population of guanacos from Torres del Paine, Chile, researchers found that population density and climate variables affected birth mass, but there was no correlation between birth mass and weather [22]. The authors suggested that the annual variation in climate could have been insufficient to influence population dynamics, and/or that population density was too high, masking any climatic influences. However, as we report here, the Tierra del Fuego guanaco population is currently fluctuating around its carrying capacity, possibly making the effects of winter temperature more conspicuous. As we mention above, further research will be needed to determine the influence on such weather variables on the demography of the species.

Studies on vicuña, a close relative of the guanaco, may shed some light on the density dependence mechanisms that regulate the Tierra del Fuego guanaco population growth. These studies suggest that density affects fecundity, which is reduced with a decrease in the amount of nutrients and water available during their first 3–4 years of life [20], [21]. Similarly, in a study of the guanaco population from Río Negro, Argentina, no yearlings (i.e. juveniles between birth and age 1 year) were observed in a breeding season after an extremely dry year [51]; however, a study designed to verify if there is a density-dependent effect on fecundity is still pending for guanacos.

Although in other ungulates usually juvenile survival decreases in response to increasing population size [52], in guanacos, and despite the negative correlation observed between mean weight of newborns and population density [22], the opposite was found; the proportional hazards model indicated that the risk of mortality of chulengos (newborns between birth and age 1 year) decreased as population density increased [23]; however, this trend may have been influenced by the effect of puma predation since the mortality risk by puma decreases when the population density increases [23]. Thus, a possible negative effect of density over the chulengo survival should not be completely dismissed.

Population size may not be controlled exclusively by either density-dependent or density-independent mechanisms, but rather a combination of both [53], and our results with the Cameron guanaco population seem to conform well to this generalization. There is still a need to carry out future controlled long-term studies to elucidate how population density, climate, predation, and even diseases, interact to determine the population dynamics of guanacos, and if these factors play a role in a possible multiple equilibrium behavior of these populations.

Our results shed light into the population dynamics of South American camelids, particularly on the strongly managed populations of guanacos of Cameron, in Chile. Due to the pronounced decline in guanaco population numbers in South America, our results may be useful to inform on management alternatives guanaco populations in Cameron and in other places.

## Supporting Information

S1 Table **Guanaco population abundance, annual precipitation and average winter temperature (June - August) used in the present analysis**.
(DOC)

S2 Table **Estimates and credibility intervals for the process and data model parameters.**
(DOC)

## References

[1] Pascual M, Kareiva P, Hilborn R (1997) The influence of model structure on conclusions about the viability and harvesting of Serengeti wildebeest. Conserv Biol 11:966–976.

[2] Patterson B, Power V (2002) Contributions of forage competition, harvest, and climate fluctuation to changes in population growth of northern white-tailed deer. Oecologia 130:62–71. 28547026 10.1007/s004420100783

[3] Augustine DJ (2010) Response of native ungulates to drought in semi-arid Kenyan rangeland. Afr J Ecol 48:1009–1020.

[4] Fujita H, Calvo J (1982) Las exportaciones de productos y subproductos de la fauna silvestre en el quinquenio 1976/1980. IDIA 397/400:1–26.

[5] Franklin WL, Bas FM, Bonacic CF, Cunazza CP, Soto VN (1997) Striving to Manage Patagonia Guanacos for Sustained Use in the Grazing Agroecosystems of Southern Chile. Wildl Soc Bull 25:65–73.

[6] The IUCN Red List of Threatened Species (2013) Available: http://www.iucnredlist.org.

[7] CITES (Convention on international Trade in Endangered Species of Wildlife Fauna and Flora)- Appendices I, II and III (2013). Available: http://cites.org/eng/app/appendices.php.

[8] Abrams PA (2009) Determining the functional form of density dependence: deductive approaches for consumer-resource systems having a single resource. Am Nat 174:321–330. 19627228 10.1086/603627

[9] Case TJ (2000) An illustrated guide to Theoretical Ecology. New York: Oxford, University Press Inc.

[10] Skogland T (1986) Density dependent food limitation and maximal production in wild reindeer herds. J Wildl Manage 50:314–319.

[11] Moussalli E, Hilborn R (1986) Optimal stock size and harvest rate in multistage life history models. Can J Fish … 43:135–141.

[12] Colchero F, Medellin RA, Clark JS, Lee R, Katul GG (2009) Predicting population survival under future climate change: density dependence, drought and extraction in an insular bighorn sheep. J Anim Ecol 78:666–673. 19245378 10.1111/j.1365-2656.2009.01528.x

[13] Ostffeld R, Canham C, Pugh S (1993) Intrinsic density-dependent regulation of vole populations. Nature 366:259–261. 8232583 10.1038/366259a0

[14] Bateman AW, Ozgul A, Coulson T, Clutton-Brock TH (2012) Density dependence in group dynamics of a highly social mongoose, *Suricata suricatta* . J Anim Ecol 81:628–639. 22117843 10.1111/j.1365-2656.2011.01934.x

[15] Koons DN, Terletzky P, Adler PB, Wolfe ML, Ranglack D, et al (2012) Climate and density-dependent drivers of recruitment in plains bison. J Mammal 93:475–481.

[16] Bårdsen B-J, Tveraa T (2011) Density-dependence vs. density-independence - linking reproductive allocation to population abundance and vegetation greenness. J Anim Ecol 81:364–376. 21985598 10.1111/j.1365-2656.2011.01913.x

[17] Serrano E, Angibault J-M, Cargnelutti B, Hewison AJM (2008) Density dependence of developmental instability in a dimorphic ungulate. Biol Lett 4:512–514. 18559310 10.1098/rsbl.2008.0221 PMC2610071

[18] Stewart K, Bowyer R, Ruess RW, Dick BL, Kie JG (2006) Herbivore optimization by North American elk: consequences for theory and management. Wildl Monog 167:1–24.

[19] Rabinovich J, Hernández M, Cajal J (1985) A simulation model for the management of vicuña populations. Ecol Modell 30:275–295.

[20] Bonacic C, Macdonald DW, Galaz J, Sibly RM (2002) Density dependence in the camelid *Vicugna vicugna*: the recovery of a protected population in Chile. Oryx 36:118–125.

[21] Shaw AK, Galaz JL, Marquet PA (2012) Population dynamics of the vicuña (*Vicugna vicugna*): density-dependence, rainfall, and spatial distribution. J Mammal 93:658–666.

[22] Sarno R, Franklin W (1999) Population density and annual variation in birth mass of guanacos in southern Chile. J Mammal 80:1158–1162.

[23] Sarno R, Franklin W (1999) Maternal expenditure in the polygynous and monomorphic guanaco: suckling behavior, reproductive effort, yearly variation, and influence on juvenile survival. Behav Ecol 10:41–47.

[24] Sarno RJ, Clark WR, Bank MS, Prexl WS, Behl MJ, et al (1999) Juvenile guanaco survival: management and conservation implications. J Appl Ecol 36:937–945.

[25] Zubillaga M, Skewes O, Soto N, Rabinovich JE (2014) Density but not climate affects the population growth rate of guanacos (*Lama guanicoe*) (Artiodactyla, Camelidae). F1000Research 2. Available: http://f1000research.com/articles/2-210#.VCnJB6P1uts.mendeley. 10.12688/f1000research.2-210.v1 PMC4149246 25187878

[26] Dennis B, Ponciano JM, Lele SR, Taper ML, Staples DF (2006) Estimating density dependence, process noise, and observation error. Ecological Monographs 76:323–341.

[27] Jacobson AR, Provenzale A, von Hardenberg A, Bassano B, Festa-Bianchet M (2004) Climate forcing and density dependence in a mountain ungulate population. Ecology 85:1598–1610.

[28] Soto NV (2010) Distribución y abundancia de la población de guanacos (*Lama guanicoe*, Muller 1776) en el área agropecuaria de Tierra del Fuego (Chile) y su relación de carga animal con la ganadería ovina. Universidad Internacional de Andalucía- Universidad de Cordoba

[29] Bank MS, Sarno RJ, Campbell NK, Franklin WL (2002) Predation of guanacos (Lama guanicoe) by southernmost mountain lions (*Puma concolor*) during a historically severe winter in Torres del Paine National Park, Chile. J Zool 258:215–222.

[30] Novaro AJ, Moraga CA, Briceño C, Funes MC, Marino A (2009) First records of culpeo (*Lycalopex culpaeus*) attacks and cooperative defense by guanacos (*Lama guanicoe*). Mammalia 73:148–150.

[31] Raedeke K (1978) El guanaco de Magallanes, Chile: distribución y biología. Chile

[32] Rabinovich JE, Capurro AF, Pessina LL (1991) Vicuña use and the bioeconomics of an Andean peasant community in Catamarca, Argentina. In: Robinson. J G., Redford KH, editors. Neotropical Wildlife use and Conservation. Chicago. pp. 337–358.

[33] Franklin W, Johnson W (1994) Hand capture of newborn open-habitat ungulates: the South American guanaco. Wildl Soc Bull 22:253–259.

[34] Tellería JL (1986) Manual para el censo de los vertebrados terrestres. Madrid, España: Editorial Raíces.

[35] Davis D, Winstead R (1987) Estimación de tamaños de poblaciones de vida silvestre. In: Mosby H, Giles RJ, Schemnitz S, editors. Manual de técnicas de gestión de vida silvestre. Maryland: The Widlife Society. pp. 233–258.

[36] Caughley G (1980) Analysis of Vertebrate Populations. John Wiley & Sons.

[37] Buckland ST, Anderson DR, Burnham KP, Laake J. (1993) Distance Sampling: Estimating Abundance of Biological Populations. London: Chapman and Hall.

[38] Buckland S, Anderson D, Burnham K, Laaker J, Borchers D, et al. (2001) Introduction to Distance Sampling: Estimating Abundance of Biological Population. Oxford University Press.

[39] Dennis B, Taper M (1994) Density dependence in time series observations of natural populations: estimation and testing. Ecol Monogr 64:205–224.

[40] Clark J, Bjørnstad O (2004) Population time series: process variability, observation errors, missing values, lags, and hidden states. Ecology 85:3140–3150.

[41] Lindén A, Mäntyniemi S (2011) Using the negative binomial distribution to model overdispersion in ecological count data. Ecology 92:1414–1421. 21870615 10.1890/10-1831.1

[42] Ver Hoef JM, Boveng PL (2007) Quasi-Poisson vs. negative binomial regression: how should we model overdispersed count data? Ecology 88:2766–2772. 18051645 10.1890/07-0043.1

[43] Metropolis N, Rosenbluth AW, Rosenbluth MN, Teller AH, Teller E (1953) Equation of State Calculations by Fast Computing Machines. J Chem Phys 21:1087.

[44] Clark JS (2007) Models for Ecological Data: An Introduction. Princeton, NJ: Princeton University Press.

[45] Gelman A, Carlin JB, Stern HS, Rubin DB (2004) Bayesian data analysis. 2nd ed. Boca Raton, Florida: Chapman & Hall.

[46] Gelfand AE, Ghosh SK (1998) Model choice: a minimum posterior predictive loss approach. Biometrika 85:1–11.

[47] Team RDC (2007) R Development Core Team (2007) R: A Language and Environment for Statistical Computing. Available: www.R-project.org.

[48] Taper M, Gogan P (2002) The northern Yellowstone elk: density dependence and climatic conditions. J Wildl Manage 66:106–122.

[49] McCullough D (1999) Density dependence and life-history strategies of ungulates. J Mammal 80:1130–1146.

[50] Bonenfant C, Gaillard J (2009) Empirical evidence of density-dependence in populations of large herbivores. Adv Ecol … 41:313–357.

[51] Rey A, Novaro AJ, Sahores M, Guichón ML (2012) Demographic effects of live shearing on a guanaco population. Small Rumin Res 107:92–100.

[52] Clutton-Brock TH, Major M, Albon SD, Guinness FE (1987) Early development and population dynamics in red deer. I. Density-dependent effects on juvenile survival. J Anim Ecol 56:53–67.

[53] Horn H (1968) Regulation of animal numbers: a model counter-example. Ecology 49:776–778.

