# Supplementary material for: Bayesian Inference on the Effect of Density Dependence and Weather on a Guanaco Population from Chile
Source: PLoS One. 2014 Dec 16;9(12):e115307. doi: 10.1371/journal.pone.0115307 (PMC4267833; doi:10.1371/journal.pone.0115307)
Supplement: S1 Table — Guanaco population abundance, annual precipitation and average winter temperature (June - August) used in the present analysis. (DOC) [file pone.0115307.s001.doc]

***Table S1.*** ***Guanaco population abundance, average annual precipitation and average winter temperature used in the present analysis.*** *Guanaco population total abundance (in units of individuals) and its lower and upper limits of the 95% confidence intervals (CI), annual precipitation (P), average winter temperature (T*°*w, months June – August), and sheep population size (data some of the data points were linearly interpolation; see text for explanation). Guanaco population values were not available for years 1986 and 1996, and were linearly interpolated.*

| Year | Guanaco Population Size | 95% Lower Limit CI | 95% Upper Limit CI | *P* | *T°w* | Sheep Population Size |
| --- | --- | --- | --- | --- | --- | --- |
| 1977 | 5372 | 5176 | 5568 | 319.8 | 1.57 | 45000 |
| 1978 | 5744 | 5541 | 5947 | 358.5 | 2.5 | 45000 |
| 1979 | 6940 | 6337 | 7543 | 346.4 | 3 | 45000 |
| 1980 | 6693 | 6128 | 7258 | 333.4 | 2.13 | 45000 |
| 1981 | 8297 | 7707 | 8887 | 399 | 2.67 | 44600 |
| 1982 | 12334 | 11539 | 13129 | 320.8 | 1.5 | 44200 |
| 1983 | 10670 | 9969 | 11371 | 285.5 | 2.87 | 43800 |
| 1984 | 19078 | 18964 | 19192 | 319 | 1.03 | 43400 |
| 1985 | 11219 | 10501 | 11937 | 341.6 | 3.1 | 43000 |
| 1986 * | 11771 | 11031 | 12510 | 388.8 | 2.3 | 42600 |
| 1987 | 12323 | 11562 | 13084 | 344.9 | 2.37 | 42200 |
| 1988 | 13027 | 12265 | 13789 | 306.4 | 2.6 | 41800 |
| 1989 | 14094 | 13253 | 14935 | 398.7 | 2.7 | 41400 |
| 1990 | 14604 | 13775 | 15433 | 517.8 | 2.47 | 41000 |
| 1991 | 17775 | 17155 | 18395 | 383.2 | 1.73 | 38000 |
| 1992 | 20774 | 20219 | 21329 | 327.1 | 1.3 | 35000 |
| 1993 | 16410 | 15560 | 17260 | 370 | 2.47 | 32000 |
| 1994 | 17626 | 16716 | 18536 | 457.9 | 2.23 | 29000 |
| 1995 | 21445 | 20153 | 22737 | 389.1 | 0.37 | 26000 |
| 1996 * | 21111 | 19752 | 22470 | 404.3 | 2.97 | 27800 |
| 1997 | 20777 | 19351 | 22203 | 408 | 2.03 | 29600 |
| 1998 | 28978 | 27749 | 30207 | 455.8 | 3.77 | 31400 |
| 1999 | 27809 | 26518 | 29100 | 300.9 | 2.27 | 33200 |
| 2000 | 28935 | 27446 | 30424 | 441.3 | 2.07 | 35000 |
| 2001 | 38841 | 37366 | 40316 | 349.1 | 2.1 | 35400 |
| 2002 | 38363 | 36810 | 39916 | 397.5 | 1.7 | 35800 |
| 2003 | 32273 | 30818 | 33728 | 440.7 | 3 | 36200 |
| 2004 | 43128 | 41089 | 45166 | 362.2 | 2.9 | 36600 |
| 2005 | 58597 | 56134 | 61060 | 374 | 1.43 | 37000 |
| 2006 | 52456 | 49820 | 55092 | 413 | 2.2 | 32000 |
| 2007 | 33125 | 31360 | 34890 | 383.7 | 2.2 | 27000 |
| 2008 | 61334 | 58546 | 64123 | 350.1 | 1.43 | 22000 |
| 2009 | 60488 | 57672 | 63304 | 351.1 | 2.67 | 29667 |
| 2010 | 44773 | 42537 | 47010 | 315.1 | 1.4 | 37333 |
| 2011 | 56973 | 54410 | 59537 | 391.9 | 1.8 | 45000 |
| 2012 | 40548 | 38266 | 42830 | 296 | 2.1 | 45000 |
