# Supplementary material for: Bayesian Inference on the Effect of Density Dependence and Weather on a Guanaco Population from Chile
Source: PLoS One. 2014 Dec 16;9(12):e115307. doi: 10.1371/journal.pone.0115307 (PMC4267833; doi:10.1371/journal.pone.0115307)
Supplement: S2 Table — Estimates and credibility intervals for the process and data model parameters. (DOC) [file pone.0115307.s002.doc]

***Table S2. Estimates and credibility intervals for the process and data model parameters.*** *The PSR column refers to the potential scale reduction values with which convergence was evaluated; values close to 1 are assumed to have properly converged*.

| **Parameter** | **Symbol** | **Mean** | **2.50% CI** | **97.50% CI** | **PSR** |
| --- | --- | --- | --- | --- | --- |
| Intercept | *β*_0_ | 0.366 | 0.336 | 0.394 | 1.02 |
| Density* | *β* _1_ | -0.408 | -0.432 | -0.383 | 1.01 |
| Temperature | *β* _2_ | 0.158 | 0.146 | 0.169 | 1.01 |
| Sheep | *β* _3_ | -0.246 | -0.276 | -0.216 | 1.02 |
| Probability | *p* | 0.501 | 0.5 | 0.502 | 1 |

**for display purposes the parameters were multiplied by 10^6^*
